# Identification of QTLs and their candidate genes for the number of maize tassel branches in F_2_ from two higher generation sister lines using QTL mapping and RNA-seq analysis

**DOI:** 10.3389/fpls.2023.1202755

**Published:** 2023-08-13

**Authors:** Sun Ruidong, He Shijin, Qi Yuwei, Li Yimeng, Zhou Xiaohang, Liu Ying, Liu Xihang, Ding Mingyang, Lv Xiangling, Li Fenghai

**Affiliations:** Special Corn Institute, Shenyang Agricultural University, Shenyang, China

**Keywords:** maize, QTL, TBN, SNP array, RNA-seq

## Abstract

Tassel branch number is an important agronomic trait that is closely associated with maize kernels and yield. The regulation of genes associated with tassel branch development can provide a theoretical basis for analyzing tassel branch growth and improving maize yield. In this study. we used two high-generation sister maize lines, PCU (unbranched) and PCM (multiple-branched), to construct an F_2_ population comprising 190 individuals, which were genotyped and mapped using the Maize6H-60K single-nucleotide polymorphism array. Candidate genes associated with tassel development were subsequently identified by analyzing samples collected at three stages of tassel growth *via* RNA-seq. A total of 13 quantitative trait loci (QTLs) and 22 quantitative trait nucleotides (QTNs) associated with tassel branch number (TBN) were identified, among which, two major QTLs, *qTBN6.06-1* and *qTBN6.06-2*, on chromosome 6 were identified in two progeny populations, accounting for 15.07% to 37.64% of the phenotypic variation. Moreover, we identified 613 genes that were differentially expressed between PCU and PCM, which, according to Kyoto Encyclopedia of Genes and Genomes enrichment analysis, were enriched in amino acid metabolism and plant signal transduction pathways. Additionally, we established that the phytohormone content of Stage I tassels and the levels of indole-3-acetic acid (IAA) and IAA-glucose were higher in PCU than in PCM plants, whereas contrastingly, the levels of 5-deoxymonopolyl alcohol in PCM were higher than those in PCU. On the basis of these findings, we speculate that differences in TBN may be related to hormone content. Collectively, by combining QTL mapping and RNA-seq analysis, we identified five candidate genes associated with TBN. This study provides theoretical insights into the mechanism of tassel branch development in maize.

## 1 Introduction

As one of the most important food crops worldwide, maize is widely used in industry, agriculture, and animal husbandry (Huang et al., 2022). Indeed, in recent decades, the demand for maize has steadily increased to meet the needs of a rapidly expanding global population and economy. As such, breeding maize varieties with optimal agronomic traits is a key objective to achieve the desired increases in yield (Wang et al., 2018). In this regard, the tassel of maize, which was domesticated from the wild ancestor teosinte, is considered an important agronomic trait (Doebley et al., 1990; Matsuoka et al., 2002; Wei et al., 2018). During growth, the ear and tassel develop simultaneously and compete for nutrients when the overall nutrient uptake of maize remains unchanged (Lambert and Johnson, 1978; Brown et al., 2011). However, appropriately reducing the tassel volume and branch number can contribute to yield increases (Brewbaker, 2015). Compared with wild-type maize, yield increases of between 5% and 19% can be obtained by using artificially emasculated strains (Hunter et al., 1969; Lambert and Johnson, 1978). Given that reducing the TBN can increase the light transmittance and photosynthetic efficiency of the upper leaves (Duncan et al., 1967; Xu et al., 2017), breeders are more inclined to select for smaller tassels, with the aim of promoting increases in yield (Gao et al., 2007). However, a larger number of tassel branches can ensure sufficient pollen production, which in turn contributes to adequate seed quantity.

TBN is a complex quantitative trait controlled by multiple genes. Previous studies have analyzed the genetics of maize tassels by constructing numerous genetic populations with germplasm materials from different backgrounds. For example, an F_2_ population comprising 6,872 individuals was constructed using the LX1 and LX2 lines for QTL mapping, resulting in the identification of *Ub4*, a potential candidate gene located on chromosome 6 (Li et al., 2019). Moreover, SICAU1212 and the maize-inbred lines 3237 and B73 were used to construct BC1S1, the subsequent analysis of which revealed 21 QTLs associated with TBN on chromosomes 2, 3, 5, and 7 (Chen et al., 2017). However, the establishment of high-density genetic maps of single-nucleotide polymorphism (SNP) markers and genome-wide association study (GWAS) analysis of natural populations provide powerful tools for the fine mapping and analysis of quantitative traits. For instance, Qin employed Mo17 as a test inbred line to conduct whole-genome association analysis and identified the tassel branch-related gene *Q^Dtbn1^* (Qin et al., 2021). Using a similar strategy, Wu identified 63 QTLs distributed on 10 chromosomes, primarily concentrated on chromosomes 1, 2, and 7, that are associated with tassel branches (Wu et al., 2016). Moreover, several SNPs associated with tassel branching have been obtained based on the GWAS analysis of 513 inbred lines using a nonparametric model (Yang et al., 2014). However, most of the QTLs identified to date have been found to have small effect values or are readily affected by environmental factors, and consequently have not been applied in breeding practices.

With the rapid development of molecular biotechnology and bioinformatics, various key genes associated with tassel branch development have been identified, and their functions have been characterized. For example, ramosal1 (*Ra1*) and *Ra2* are transcription factors, whereas *Ra3* encodes a trehalose 6-phosphate phosphatase (TPP), and it has been established that *Ra2* and *Ra3* promote the expression of *Ra1*. Moreover, it has been observed that *ra1*, *ra2*, and *ra3* are associated with an increased TBN phenotype (Vollbrecht et al., 2005; Bortiri et al., 2006; Satoh-Nagasawa et al., 2006; Claeys et al., 2019). Genes from different transcription factor families are also involved in the regulation of TBN, notable among which is barren stalk 1 (*Ba1*), which encodes a basic helix-loop-helix (bHLH) transcription factor that influences TBN by regulating meristem transformation processes (Gallavotti et al., 2004). The ethylene response factor (ERF) family encoding the APETALA2 (AP2) transcription factor indeterminate spikelet 1 (*Ids1*) and sister of indeterminate spikelet 1 (*Sid1*) has also been demonstrated to regulate tassel development (Chuck et al., 1998; Chuck et al., 2008). Furthermore, three genes, namely, tassel sheath 4 (*Tsh4*), unbranched 2 (*Ub2*), and *Ub3*, belonging to the squamosa promoter binding-box transcription factor family, have been found to contribute to TBN regulation. Notably, these three genes are characterized by functional redundance, with single, double, and triple mutant plants showing marked reductions in TBN and an increase in the number of rows of spikes (Chuck et al., 2014). In addition, mutants of the gene liguleless 2 (*Lg2*), which regulates leaf angle, can also be characterized by lower TBNs (Walsh et al., 1998; Walsh and Freeling, 1999).

TBN development is also regulated by different plant hormones, including auxins, cytokinins (CKs), and strigolactones (SLs) (Isbell and Morgan, 1982; Ongaro and Leyser, 2008; Umehara et al., 2008; McSteen, 2009). Among these, auxins are synthesized in the shoot apical meristem (SAM) and transported downward by polar auxin transport, thereby inhibiting branch formation and inducing apical dominance. Contrastingly, CKs are synthesized in roots and stems and promote the synthesis of auxins and, thus, the development of collateral branches (Mueller and Leyser, 2011). CKs also regulate apical meristem size, whereas a loss of function of the lonely guy (Log) and wuschel (*Wus*) genes influences CK synthesis and transport, leading to early SAM termination, and modification of TBN development (Ongaro and Leyser, 2008; Umehara et al., 2008). As carotenoid-derived plant hormones, SLs are also involved in the regulation of branching. For instance, transgenic corn plants overexpressing maize *Dwarf 53* (*ZmD53*) are characterized by excessive tillering and reduced TBN, whereas *ZmD53* interacts with the SL receptor *ZmD14A/B* in a rac-Gr24-dependent manner (Liu et al., 2021). In this way, SLs influence auxin transport by regulating auxin export carrier proteins, thereby leading to altered TBN (Ongaro and Leyser, 2008; Durbak et al., 2012).

To gain further insights into the genetic regulation of maize TBN, in this study, we employed the Maize6H-60K gene array to produce a high-density genetic linkage map of the F_2_ population generated using two sister lines, namely the unbranched inbred line, PCU, and multi-branched inbred line, PCM. Subsequently, the genetic linkage map and two-year phenotypic data were used to map QTLs associated with TBN. By analyzing the RNA-seq data, we compared the changes in gene expression between the two parents at different stages of tassel development. Furthermore, the results of QTL mapping and RNA-seq analysis were combined to screen for candidate genes regulating TBN. Our findings in this study can be used as a reference for verifying the function of genes associated with TBN and provide a theoretical basis for genetic improvement of the maize tassel branch trait and associated molecular breeding.

## 2 Materials and methods

### 2.1 Plant materials and construction of mapping populations

The sister lines PCU and PCM were bred using the parents Xianyu 335 and Zheng 58, in which PCU was the non-branching material (TBN, 0) and PCM was the multi-branched material (TBN, 5–8), both of which were provided by the Special Maize Research Institute of Shenyang Agricultural University (Liaoning, China). A total of 994 pairs of simple sequence repeat (SSR) markers and SNP markers were used to assess PCU and PCM, which were established to have a genetic similarity of 93.17%. Subsequently, a single F_2_ population comprising 190 plants was developed by crossing PCU and PCM within the experimental field of Shenyang Agricultural University (Shenyang, Liaoning, 41.48°N, 123.25°E). The F_2:3_ population was planted at the Southern Breeding Base of Shenyang Agricultural University (Sanya, Hainan, 18.15°N, 109.30°E). The width and length of the single-row plot were 65 cm and 4 m, respectively, and the spacing between the plants was 20 cm, according to standard field management methods.

### 2.2 Determination and analysis of phenotype data

After the maize tassels had matured, we investigated the TBN phenotypes, with branches bearing more than one pair of small flowers being considered effective branches. The average branching number was used as the phenotype data for the F_2:3_ population. The statistical parameters of TBN in the F_2_ and F_2:3_ populations were calculated using SPSS software version 24.0. Pearson correlation coefficients and phenotype frequency distribution maps were visualized using the R package ggpubr performance analytics.

### 2.3 Genetic mapping and QTL and QTN detection

The parent plants and 190 F_2_ individuals were genotyped using a Maize6H-60K SNP array (Tian et al., 2021). Linkage analysis was performed using QTL ICIMAPPING 4.2 software (Meng et al., 2015), in which markers with no polymorphism between parents and a deletion rate > 10% were removed. The TBN was assessed using the inclusive composite interval mapping method (ICIM) in the software QTL ICIMAPPING 4.2 (Meng et al., 2015), composite interval mapping method (CIM) in the Windows QTL Cartographer 2.5 (Wang et al., 2012), and genome-wide composite interval mapping (GCIM) (https://cran.r-project.org/web/packages/QTL.gCIMapping/index.html) (Wen et al., 2019) and dQTG-seq2 (https://cran.r-project.org/web/packages/dQTG.seq/index.html) (Li et al., 2022). QTLs were evaluated based on 1,000 permutation tests with a significance level of 0.05 to determine the logarithm of the odds (LOD) threshold and thereby identify QTLs. A slightly more stringent criterion (*P*-value = 0.00316) was applied to denote significant QTLs, which was converted from an LOD score of 2.50. When adopting the dQTG-seq2 method, we used the 20% plants with the highest TBN as the high pool and the 20% of plants with the lowest TBN as the low pool.

### 2.4 RNA isolation and RNA-seq

The tissues of PCU and PCM tassels collected at three different stages of development, namely, the growth cone elongation stage (Stage I), the early stage of tassel differentiation (Stage II), and the later stage of tassel differentiation stage (Stage III), were immersed in an RNA storage solution (Li et al., 2019). PCU and PCM had similar tassel-branching stem tips during Stage I. However, it is uncertain as to whether the lateral meristems differentiated into tassel branches during Stage II. During Stage III, tassel branches at the base of PCU and PCM could be clearly distinguished. RNA extraction was performed using the TRIzol method (Rio et al., 2010).

For each line at each stage, we obtained three duplicate biological samples, and used the total 18 samples to construct a cDNA library. Construction and sequencing of the library were performed by Beijing Nohezhiyuan Bioinformation Technology Co., Ltd (Tianjin). Using an Illumina Hiseq™4000 high-throughput sequencing platform to obtain 100-bp double-terminal sequence reads, and FastQC tools (http://www.bioinformatics.babraham.ac.uk/projects/fastqc/) was used to control the read quality. Low-quality reads were removed using Trimmomotic 0.36 (Bolger et al., 2014). The reference genome (AGPv4) was obtained from the maize database MaizeGDB (https://maizegdb.org). To calibrate the FastQC output, gene expression levels were normalized based on gene length and the number of reads, and the number of transcription fragments per kilobyte/million mapping reads (FPKM) was calculated. The DESeq software package was used to identify those genes that were differentially expressed (DEGs) between PCU and PCM (Anders and Huber, 2010).

Functional annotation and Gene Ontology (GO) analysis of genes were performed using Blast2go 4.1 (Conesa et al., 2005). whereas Kyoto Encyclopedia of Genes and Genomes (KEGG) Orthology-based Annotation System KOBAS 2.0 software (http://kobas.cbi.pku.edu.cn) was used to perform pathway enrichment analysis. The *P*-value of each gene was adjusted using the Benjamini and Hochberg method to control the false discovery rate. *P*-values < 0.05 and | log2FC | ≥ 1 were applied as thresholds to identify DEGs.

Venn diagrams are drawn by online sites. (https://bioinfogp.cnb.csic.es/tools/venny/index.html)

### 2.5 qRT-PCR

RNA derived from tassels collected at the three stages (Stage I, II, and III) was assessed *via* qRT-PCR, for which primers were designed using Primer BLAST (https://www.ncbi.nlm.nih.gov/tools/primer-blast) (**Table S8**). All primers were synthesized and supplied by Shenggong Biotech Co., Ltd. The housekeeping gene *Gapdh* was used as the internal reference gene, the relative expression levels of which were calculated using the 2^-ΔΔct^ method.

### 2.6 Determination of hormone content

Tassels collected at Stage I were exfoliated, flash frozen in liquid nitrogen, and stored at -80°C. A standard plant hormone solution was prepared using a 50% formaldehyde solution, and 10 μL of an internal standard plant hormone solution was added to 50 μL of a concentration gradient of standard plant hormone solutions. Thereafter, 1 mL of methanol/water/formic acid mixture (15:4:1, v/v/v) was added, followed by vortexing for 10 min (until thoroughly mixed), and the resultant mixture was allowed to stand for 12 h. The auxin, CK, ethylene (ETH), abscisic acid (ABA), gibberellin (GA), and SL contents of the tassels were determined by analyzing the resultant supernatant *via* liquid chromatography in conjunction with tandem mass spectrometry (LC-MS/MS).

### 2.7 Identification of candidate genes

Genes located in the vicinity of large loci with an R^2^ value > 10%, and which were stable across 2 years, were used for gene annotation. Gene annotation information was obtained using MaizeGDB (https://maizegdb.org) and Phytozome (http://phytozome.jgi.doe.gov). Gene expression in PCU and PCM was analyzed using RNA-seq data and applied to predict gene function that might be associated with tassel branching in maize.

### 2.8 Cloning and sequence alignment of Zm00001d038537

The candidate gene *Zm00001d038537* was extracted from the genomic DNA and cDNA of PCU and PCM. The primers used for amplification are listed in **Supplementary Table S8**. DNA sequence alignment was performed using SnapGene software (https://www.snapgene.com/).

## 3 Results

### 3.1 Statistical differences in plant architectural traits and phenotypic analysis in sister lines

Architectural traits of plants of the sister lines PCU and PCM were compared and analyzed. Apart from leaf length, leaf width, leaf angle, and TBN, we detected no significant differences between the two lines with respect to plant architecture (**Table 1**). Notably, over the 2 years of the study, we detected a significant difference between the parent lines with respect to TBN, with PCM being characterized by a larger number of tassel branches, whereas under certain environmental conditions, PCU had no branches, thereby indicating that these phenotypic traits of the parents are probably stable (**Table 2**).

**Table 1:** Statistical difference of agronomic traits in sister lines.

Traits	PCU		PCM	
	Mean	SD *^a^*	Mean	SD *^a^*
Plant height(cm)	223.4	4.4	220.1	3.7
Ear height(cm)	88.4	2.1	85.7	2.4
Leaf angle(°)	31.2	3.0	67.7**	4.0
Leaf length(cm)	76.3	3.0	66.1**	3.0
Leaf width(cm)	10.9	0.4	8.6**	0.5
TBN	0.0	0.0	5.1**	1.2
Stem diameter(mm)	26.5	2.0	25.6	1.9
Ear length(cm)	15.7	1.1	15.4	0.9
Ear diameter(mm)	36.9	0.9	36.4	0.7
Ear rows	14.0	0.0	14.0	0.0
Hundred grain weight(g)	26.7	1.1	25.3	0.9

^a^ SD, Standard Deviation. The asterisks (*or **) represent the significant differences at P < 0.05 or P< 0.01, respectively.

**Table 2:** Mean, extreme, Standard Deviation (SD), Coefficient of Variation (CV), Skewness and Kurtosis of the TBN in parents and F_2_, F_2:3_ populations.

Parents			offspring of PCU×PCM						
	PCU *^a^*	PCM *^a^*	Min	Max	Mean	SD *^b^*	CV (%) *^c^*	Skewness	Kurtosis
F_2_	0	5.1	0	11	2.55	2.54	99.65	0.97	0.36
F_2:3_	0	4.9	0	5.43	1.71	1.32	76.92	0.52	-0.57

^a^ Mean TBN of PCU and PCM calculated from 10 plants per parent in two rows. ^b^ SD, Standard Deviation; ^c^ Coeﬃcient of Variation.

**Figure 1:**
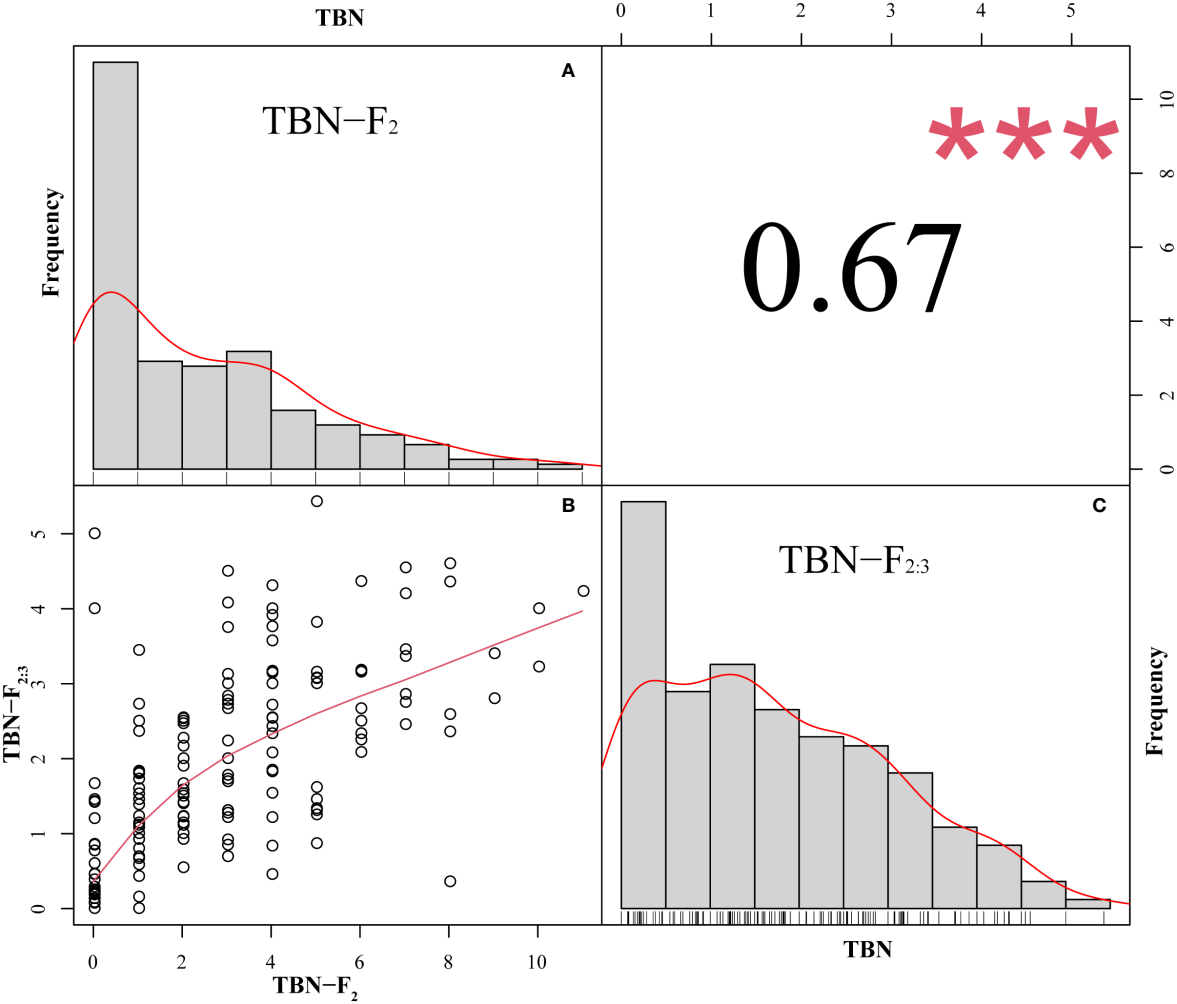
Frequency distribution and correlation of TBN of F_2_ and F_2:3_, ***P<0.001. **(A)** The horizontal coordinate is TBN and the vertical coordinate is the frequency. **(B)** The TBN distribution of the F_2_ population in the horizontal coordinate and the F_2:3_ population in the vertical coordinate. **(C)**: The horizontal coordinate is TBN and the vertical coordinate is the frequency.

The TBN of the F_2_ population ranged from 0 to 11, with a coefficient of variation of 99.65%, whereas in the F_2:3_ population, the TBN ranged from 0 to 5.43, with a coefficient of variation of 76.92%. In both offspring populations, the number of tassel branches was maintained at an average of that of the two parents (**Table 2**). Moreover, we detected a highly significant correlation between F_2_ and F_2:3_. The TBN of the two offspring groups was biased toward PCU and exhibited a continuous distribution trend (**Figure 1**). In addition, the skewness and kurtosis results revealed that both populations conformed to the quantitative trait characteristics of skewed normal distribution and polygene control (**Table 2**). Accordingly, the two progeny populations were assumed to meet the requirements for QTL mapping.

### 3.2 QTL and QTN identification and effect calculations

**Figure 2:**
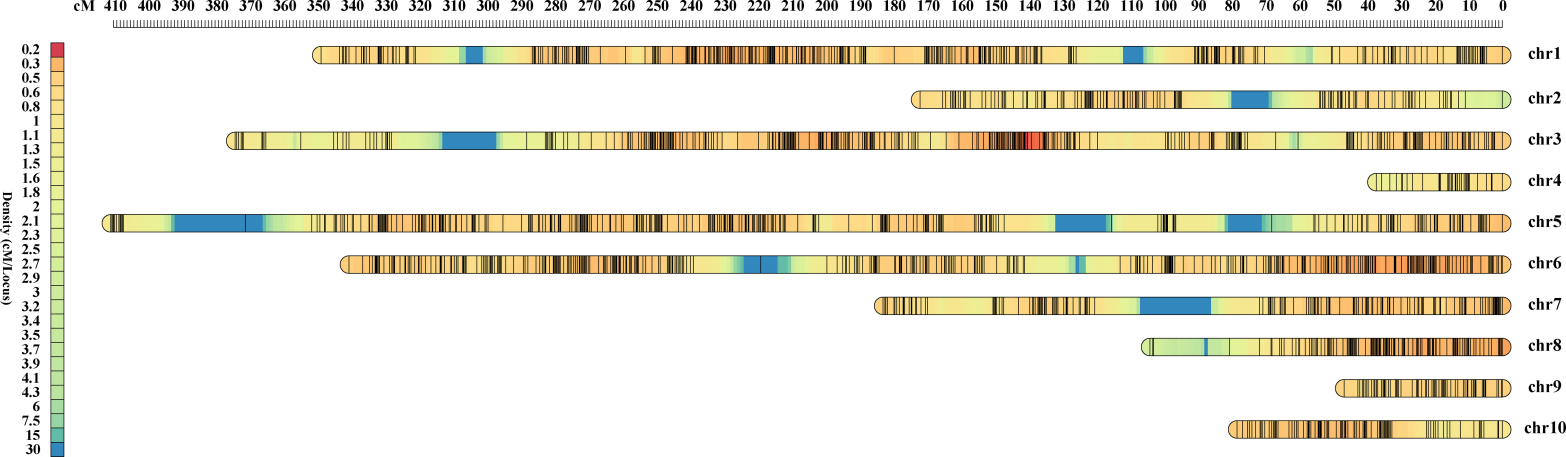
Genetic map of F_2_ population. The upper ruler shows the distance between SNP markers in centimorgans (cM), the color shades of the left ruler represent the density of SNP markers on each linkage group.

The F_2_ population was genotyped using the Maize6H-60K SNP array, which contains 61,214 SNP markers covering the entire maize genome. A genetic linkage map was constructed by screening high-quality genotype-independent SNP markers with deletion rates < 10% between the two parents, from which we obtained 4,136 SNP markers (**Table S1**). The linkage map covered a distance of 2,095.02 cM, with an average distance of 0.51 cM between markers. The number of SNP markers on each chromosome ranged from 46 to 710, with a linkage distance ranging from 37.22 to 410.78 cM (**Table 3**). As the two parents are higher generation sister lines with high background similarity, the SNP differences detected on chromosomes 4 and 9 were small (**Figure 2**).

**Table 3:** Total SNP numbers and linkage distances of chromosomes in F_2_ population.

Chromosome	Number of SNPs	Linkage Distance(cM)	Average Distance between Markers(cM)
1	726	348.72	0.48
2	235	171.88	0.73
3	759	374.11	0.49
4	46	37.22	0.81
5	678	410.78	0.61
6	809	340.48	0.42
7	341	182.79	0.54
8	249	103.97	0.42
9	103	46.73	0.45
10	190	78.34	0.41
Total	4136	2095.02	0.51

Combined with phenotype data of the two populations and the F_2_ genetic linkage map, QTLs for the TBN of F_2_ and F_2:3_ were identified using ICIM, CIM, and GCIM methods. Within the two populations, we detected 13 QTLs associated with TBN on chromosomes 3, 6, and 7, with LOD values ranging from 5.10 to 40.78 and accounting for 6.86% to 37.64% of the phenotypic variation (**Table 4**; **Figure S4**). Excluding *qTBN-3-4* and *qTBN-3-5*, which exhibited a positive additive effect attributable to the PCM allele, the other QTL sites showed negative additive effects associated with the PCU allele. In addition, we identified 22 SNPs significantly associated with TBN based on dQTG-seq2 mapping. Compared with other methods, we identified new SNPs on chromosomes 1, 2, 4, and 5 when using dQTG-seq2. The upstream and downstream 50 kb of the significantly associated SNPs were used as the intervals for predicting candidate genes (**Table 5**; **Figure S5**) (Li et al., 2013).

**Table 4:** Analysis of TBN-related QTLs in offspring population from PCU×PCM.

QTL *^a^*	Chromosome	Mapping interval/bp *^b^*	Position	LOD *^c^*	Additive effect	Dominant effect	R^2^(%) *^d^*	Generation	Method
*qTBN-3-1*	3	179394655-179625328	96	6.59	-0.96	0.05	9.25	F_2_	ICIM
	3	179392238-179900293	96	6.40	-0.95	0.09	6.98	F_2_	GCIM
*qTBN-3-2*	3	134150716-178936874	98	6.37	-1.24	0.10	7.84	F_2_	CIM
*qTBN-3-3*	3	182413848-182508246	48	6.80	-0.40	0.15	6.80	F_2:3_	ICIM
*qTBN-3-4*	3	2019660-2050620	333	8.35	0.03	0.62	8.34	F_2:3_	ICIM
*qTBN-3-5*	3	1473821-1548536	353	5.39	0.09	-0.49	5.39	F_2:3_	ICIM
*qTBN-6-1*	6	157846342-159598073	235.9	38.89	-1.47	-0.23	37.64	F_2:3_	CIM
	6	157846342-159598073	237	40.78	-1.27	-0.08	40.77	F_2:3_	ICIM
	6	159231856-159316218	240	20.83	-1.91	-0.30	34.63	F_2_	ICIM
	6	159231856-159316218	240	15.46	-1.88	-0.27	27.63	F_2_	GCIM
	6	159116395-159231856	242.5	33.35	-1.27	-0.05	34.81	F_2:3_	CIM
	6	159141240-159355691	243.8	15.62	-1.87	-0.27	15.07	F_2_	CIM
	6	159355691-159538438	244.8	4.62	-0.64	-0.68	18.40	F_2:3_	GCIM
*qTBN-6-2*	6	159648428-159792909	246.5	33.84	-1.36	0.00	37.02	F_2:3_	CIM
*qTBN-6-3*	6	160665895-160691260	253	5.04	-0.93	-0.82	34.54	F_2:3_	GCIM
*qTBN-6-4*	6	160691260-160895678	254.7	11.03	-1.80	-0.38	9.39	F_2_	CIM
	6	160691260-160895678	254.7	28.63	-1.38	-0.13	28.94	F_2:3_	CIM
*qTBN-6-5*	6	168094283-168363228	307	4.77	-0.80	-0.43	4.61	F_2_	GCIM
	6	168200733-168363228	318	5.10	-0.77	-0.35	6.86	F_2_	ICIM
	6	169161160-169372663	319	6.52	-0.37	0.00	6.52	F_2:3_	ICIM
*qTBN-7-1*	7	127691371-128260837	149	5.39	-0.77	-0.37	7.34	F_2_	ICIM
	7	127691371-128260837	149	4.77	-0.72	-0.40	4.61	F_2_	GCIM
*qTBN-7-2*	7	123889115-125102662	145	5.42	-0.32	0.02	5.42	F_2:3_	ICIM
*qTBN-7-3*	7	128260837-172487130	111.8	5.50	-0.47	-0.15	4.63	F_2:3_	CIM
	7	125921578-127728775	128.6	5.38	-0.37	0.03	4.36	F_2:3_	CIM

^a^QTL detected in different methods and generations at the same, adjacent, or overlapping marker intervals was considered as the same QTL. ^b^Physical position of the 95% confidence interval for the detected QTL. ^c^LOD (Logarithm of odds) value at the peak likelihood of the QTL. ^d^Phenotypic variance (R^2^) explained by the detected QTL.

**Table 5:** Significant QTNs for TBN in F_2_ and F_2:3_ using dQTG-seq2 method.

Generation	Maker	Chromosome	Position	Mapping interval/bp	Gw *^a^*	Smooth_Gw *^b^*
F_2_	AX-108052314	1	227992408	227942408-228042408	6.91	7.78
	AX-108019986	3	178936874	178886874-178986874	7.52	8.43
	AX-107939474	3	180214656	180164656-180264656	10.77	10.31
	AX-86317565	6	159792909	159742909-159752909	102.67	102.02
	AX-91021926	6	172603449	172553449-172653449	17.68	18.41
F_2:3_	AX-247233306	2	223266472	223176472-223276472	9.03	9.81
	AX-107941057	3	110309750	110259750-110359750	8.41	8.85
	AX-108009558	3	117879603	117829603-117929603	8.84	8.68
	AX-108061753	3	130582492	130532492-130632492	10.67	9.7
	AX-90827906	3	132093889	132043889-132143889	9.84	9.73
	AX-108019986	3	178936874	178886874-178986874	11.11	17.48
	AX-247236770	4	824775	774775-874775	10.9	10.65
	AX-107945551	4	3672068	3622068-3722068	8.29	9.15
	AX-178079230	5	7392849	7342849-7352849	10.66	9.24
	AX-107981631	5	212584879	212534879-212634879	9.47	12.15
	AX-107989634	5	222130069	222080069-222180069	10.13	10.07
	AX-108011870	6	150255513	150205513-150305513	27.67	26.13
	AX-91016539	6	153616434	153566434-153666434	16.88	14.1
	AX-86317565	6	159792909	159742909-159752909	62.31	64.83
	AX-86294633	6	163542081	163492089-163592089	59.83	58.99
	AX-86301494	6	166848539	166798539-166898539	24.81	24.47
	AX-91021926	6	172603449	172553449-172653449	17.57	18.92

^a^ Gw: The value of statistic Gw calculated by the dQTGseq2 method. ^b^ Smooth_Gw: smooth Gw value of one marker via the window size method.

On the basis of statistical analysis of QTLs and QTNs, we identified two QTLs on chromosome 6 with R^2^ > 10%, namely, *qTBN6.06-1* (157846342–159598073 bp) and *qTBN6.06-2* (159648428–159792909 bp) (**Table 4**). Moreover, we identified candidate genes in the two QTLs based on the physical location of the SNP markers. *qTBN6.06-1* and *qTBN6.06-2* contained 73 and 14 genes, respectively (**Table S2**). In contrast to the findings of previous studies, we failed to identify any TBN-related genes in *qTBN6.06-1* and *qTBN6.06-2*. Hence, we used the online tool Web Gene Ontology Annotation Plot (WEGO) 2.0 (Ye et al., 2018) to annotate the candidate genes within the two QTL intervals. The results revealed that binding (GO:0005488), metabolic process (GO:0008152), and cellular process (GO:0009987) were the three main GO entries for the 84 genes in the two QTLs (**Figures S1**; **S2**), and consequently, we speculate that tassel development is associated with these processes.

### 3.3 RNA-seq analysis

**Figure 3:**
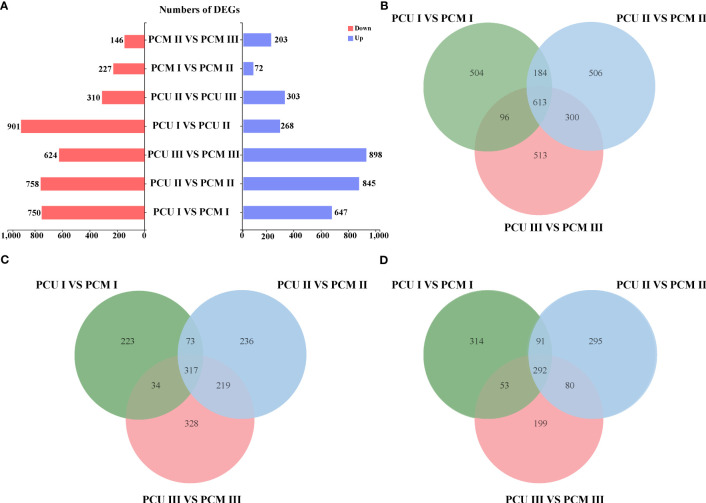
Numbers of PCU and PCM differentially expressed genes in Stage I, II and III. **(A)** Comparison of the number of differentially expressed genes in different stages and between different parents. **(B)** Number of co-differentially expressed genes in Stage I, Stage II, and Stage III. **(C)** The number of genes is co-upregulated in three stages. **(D)** The number of genes is co-downregulated in three stages.

**Figure 4:**
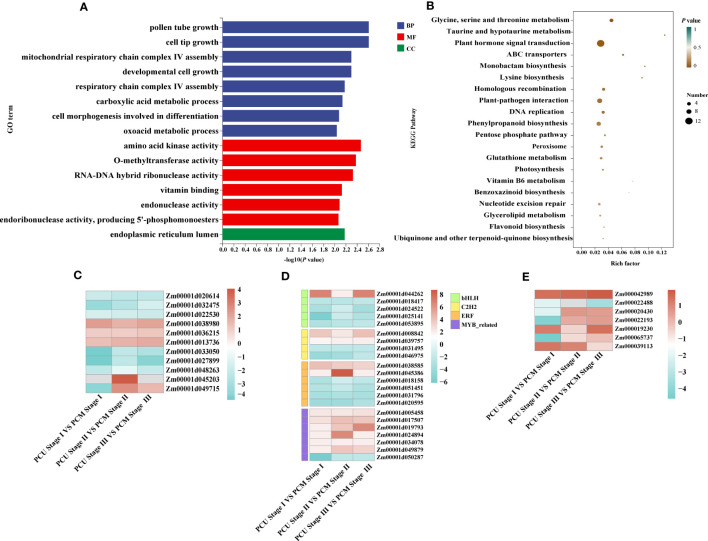
GO and KEGG analysis and changes in the expression levels of differentially expressed genes (DEGs). **(A)** GO enrichment analysis was executed with DEGs identified between PCU and PCM. The ordinate and abscissa represent the main biological process GO terms and -Log10(*P*-value), respectively. **(B)** KEGG enrichment analysis was executed with DEGs identified between PCU and PCM. The ordinate and abscissa represent the major KEGG biological pathways and rich factor, the size of the dots represents the number of genes enriched, respectively. **(C)** Expression levels of plant hormone signal transduction pathway-related genes. **(D)** Gene expression levels of different transcription factor families. **(E)** The expression levels of genes related to tassel development are known. The value is the log2 fold-change (log2(FC)) of each gene. The colors of the boxes represent upregulated (red) and downregulated (blue) genes.

Despite our GO enrichment analysis of genes within the localized intervals, differences in gene expression during tassel development remained undetermined. Consequently, to identify the genes responsible for tassel branch development, we compared the DEGs (|Log2-fold change| ≥ 1 and *P*-value < 0.05) between PCU and PCM at the three assessed developmental stages. We analyzed DEGs common to Stages I, II, and III, among which, 317 and 292 genes were up- and downregulated, respectively (**Figures 3A–D**; **Table S3**). GO enrichment analysis revealed a significant enrichment of 118 biological processes (**Table S4**), which are primarily associated with the growth and development of tissues or cells, including pollen tube growth, cell tip growth, amino acid kinase activity, developmental cell growth, and the endoplasmic reticulum lumen (**Figure 4A**). In addition, we identified enrichment of several pathways associated with enzyme activity, including those of endonuclease, endoribonuclease, mitogen-activated protein (MAP) kinase, inositol-3-phosphate synthase, and glyceraldehyde-3-phosphate dehydrogenase (NADP+) (phosphorylating). Therefore, we speculate that the activities of different enzymes also influence TBN.

KEGG enrichment analysis further revealed that DEGs were enriched in glycine, serine, and threonine metabolism; taurine and taurine metabolism; plant hormone signal transduction; ATP-binding cassette (ABC) transporter superfamily (**Figure 4B**; **Table S6**). In maize, BARREN INFLORESCENCE2 (*Bif2*) encodes a serine/threonine protein kinase *Bif2* phosphorylates *ZmPIN1a*, *Bif2* regulates auxin transport through direct regulation of *ZmPIN1a* during maize inflorescence development (Skirpan et al., 2009; Forestan et al., 2012). The main functions of ABCB protein in ABC transporter family are auxin transport. In *Arabidopsis thaliana* studies, it was found that *ATABCB1*, *ATABCB6*, ATABCB14, *ATABCB15* and *ATABCB20* all participated in auxin transport in inflorescence axis, which further affected the growth and development of inflorescence axis (Okamoto et al., 2016). Thus, the above pathways may be involved in TBN development. Among these, 12 genes were enriched in plant hormone signaling pathways, seven of which were associated with indole-3-acetic acid (IAA) signaling. Other pathways were primarily associated with amino acid anabolism. Accordingly, KEGG pathway analysis provided evidence to indicate that tassel branch development might be associated with hormone and energy metabolism (**Figure 4C**).

Simultaneously, we annotated the DEGs, on the basis of which we retrieved 64 transcription factors, among which myeloblastosis (MYB)-related genes (seven) were the most common, followed by ERF (six), bHLH (five), and C2H2 (Cys2/His2-type; four) genes. In addition, we also identified three auxin response factors (ARFs). Interestingly, the expression of most MYB-related genes in PCU was higher than that in PCM, whereas ERF transcription factor expression was downregulated in PCM (**Figure 4D**; **Table S7**).

We also performed GO enrichment analysis for genes differentially expressed in only one of the three assessed stages. Those exclusively identified in Stage I were primarily enriched in the regulation of nitrogen compound metabolic processes, regulation of primary metabolic processes, and regulation of nucleic acid-templated transcription, which are closely associated with plant growth and development (**Table S5**). Moreover, certain genes known to regulate tassel development in maize were analyzed (**Figure 4E**), most of which were differentially expressed in Stage I, with the variance fold change being greater than that in the other two stages. On the basis of these findings, we assume that Stage I is critical to the regulation of tassel development.

### 3.4 Determination of hormone content

**Figure 5:**
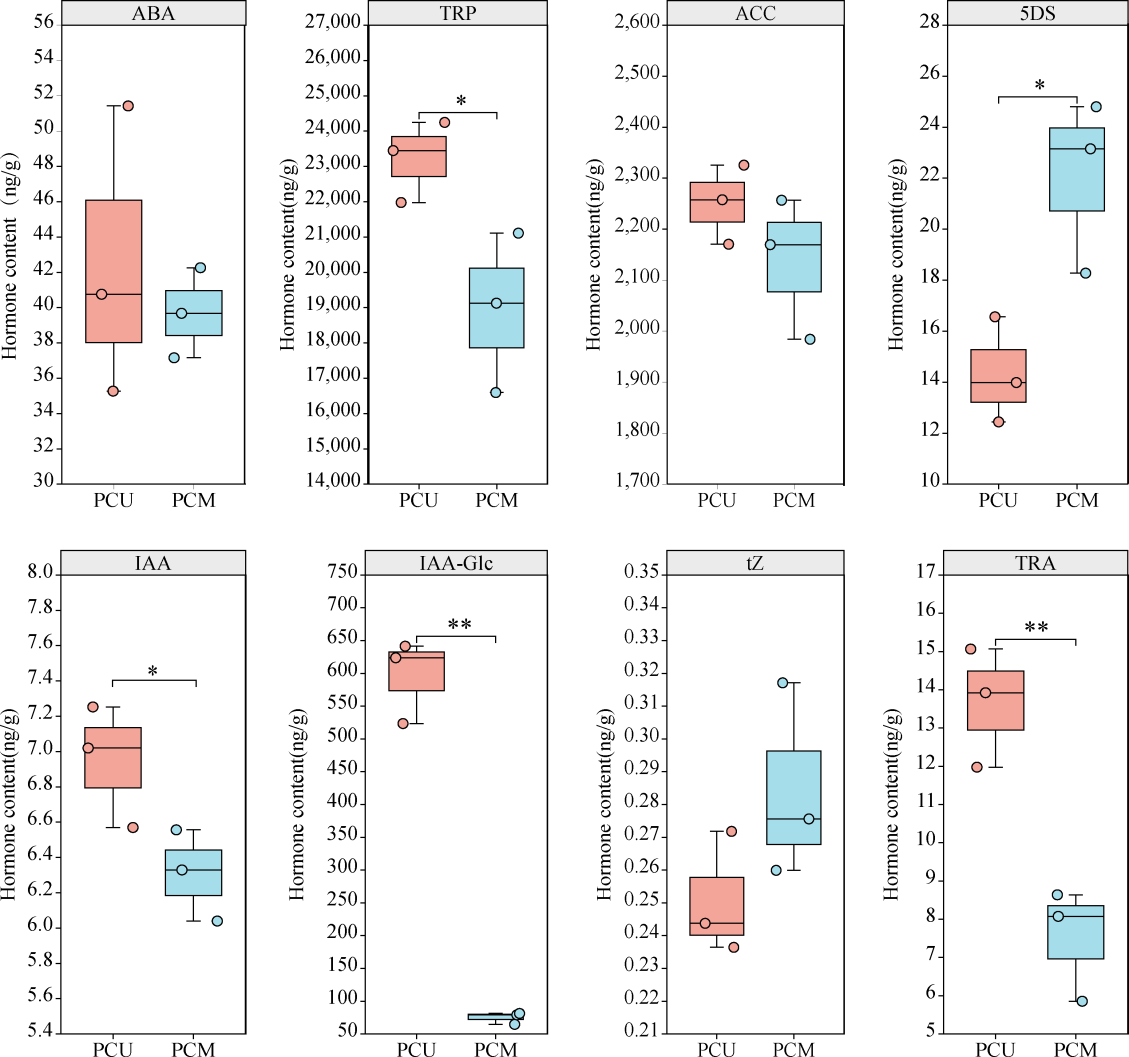
Different phytohormone contents of PCU and PCM in Stage I. The asterisks (*or **) represent the significant differences at *P* < 0.05 or *P* < 0.01, respectively.

Our KEGG results provided evidence to indicate that DEGs were enriched in plant hormone signal transduction, and we speculated that Stage I was the key stage responsible for the observed differences between PCU and PCM with respect to TBN. We thus used samples of Stage I PCU and PCM tassels to quantify hormone content, which revealed that the content of IAA in PCU was slightly higher than that in PCM, whereas the respective contents of tryptamine (TRA) and tryptophan (TRY), two important precursors in the auxin synthesis pathway, were significantly higher in PCU. In addition, the content of IAA-glc, an important form of stored IAA. was found to be 7.9-fold higher in PCU than in PCM, whereas in contrast, the content of 5-deoxymonopolyl alcohol (5DS), the first active product of the SL biosynthetic pathway, was found to be significantly higher in PCM than in PCU. However, we detected no significant differences between the lines with respect to the levels of ABA, trans-zeatin (tZ), or 1-aminocyclopropanecarboxylic acid (ACC). On the basis of these observations, we can speculate that differences in the tassel branching phenotypes of the two parent lines are attributable, at least in part, to differences in the contents of IAA and 5DS (**Figure 5**).

### 3.5 Predicting candidate genes

To screen for candidate genes, we selected 614 common DEGs to cross-analyze the mapping interval. The interval *qTBN6.06-1* comprised 73 protein-coding genes, 27 of which were negligibly expressed during the three stages of tassel development, and 38 showed no significant differences. Only two genes, *Zm00001d038519* and *Zm00001d038523*, were differentially expressed at all three stages. Of the 14 protein-encoding genes present within *qTBN6.06-2*, only *Zm00001d038546* and *Zm00001d038552* were identified as being differentially expressed during the three stages.

**Figure 6:**
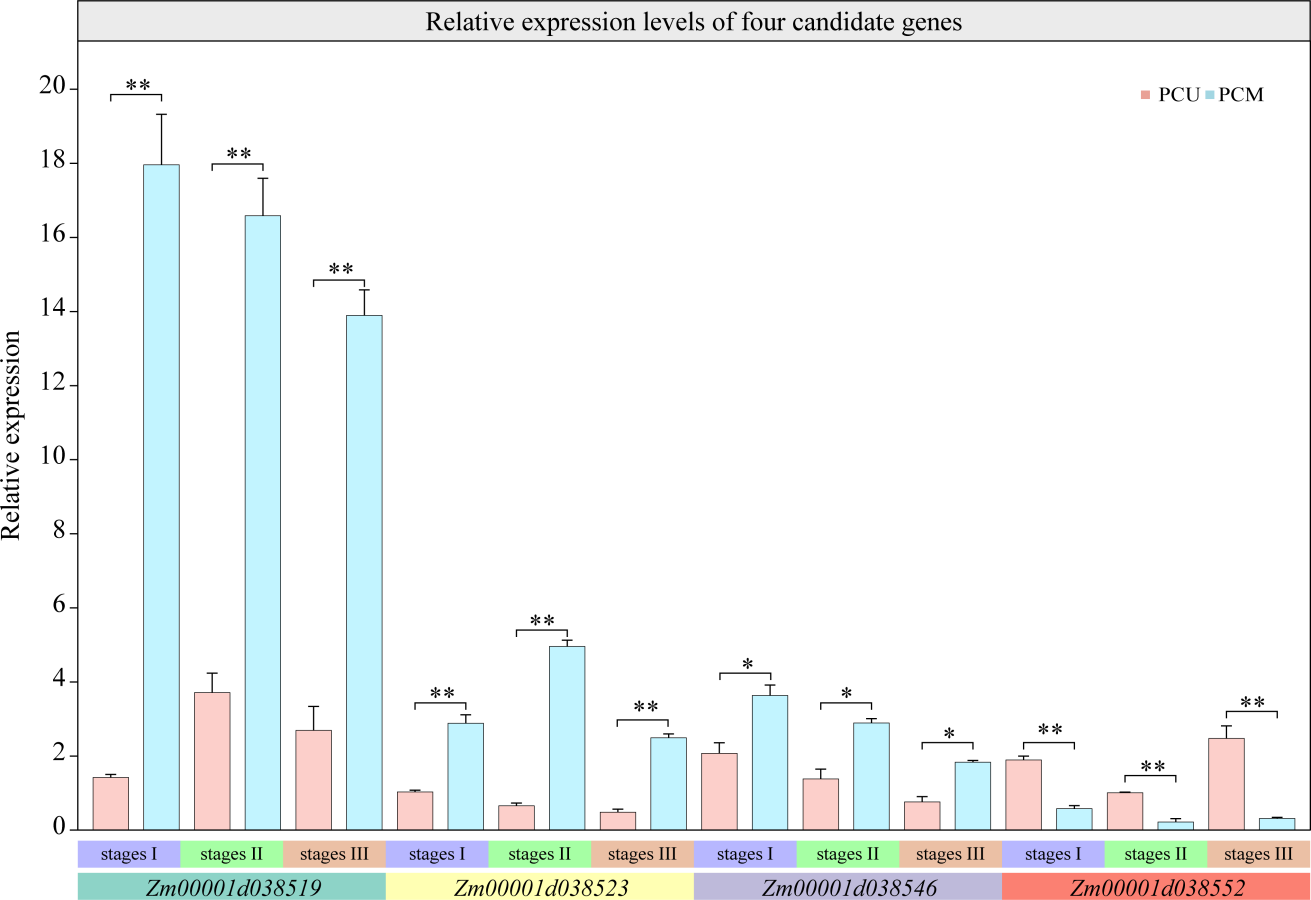
Relative expression levels of four candidate genes at three stages analyzed *via* qRT-PCR. The asterisks (*or **) represent the significant differences at *P* < 0.05 or *P* < 0.01, respectively.

These four candidate genes were annotated using Phytozome (https://phytozome-next.jgi.doe.gov/), using which, *Zm00001d038519* was predicted to contain a putative S-adenosyl-L-methionine-dependent methyltransferase domain, which regulates plant growth and development *via* methylation. We thus inferred that *Zm00001d038519* might have a similar function. *Zm00001d038546* was found to contain a Myb-like DNA-binding domain and thus could be a member of the MYB family of transcription factors that are primarily involved in inflorescence development and the segregation of lateral organs. However, using this approach, we were unable to predict structures for *Zm00001d038523* or *Zm00001d038552*. The four candidate genes were verified *via* qRT-PCR analysis, and the results were consistent with those obtained based on RNA-seq (**Figure 6**).

**Figure 7:**
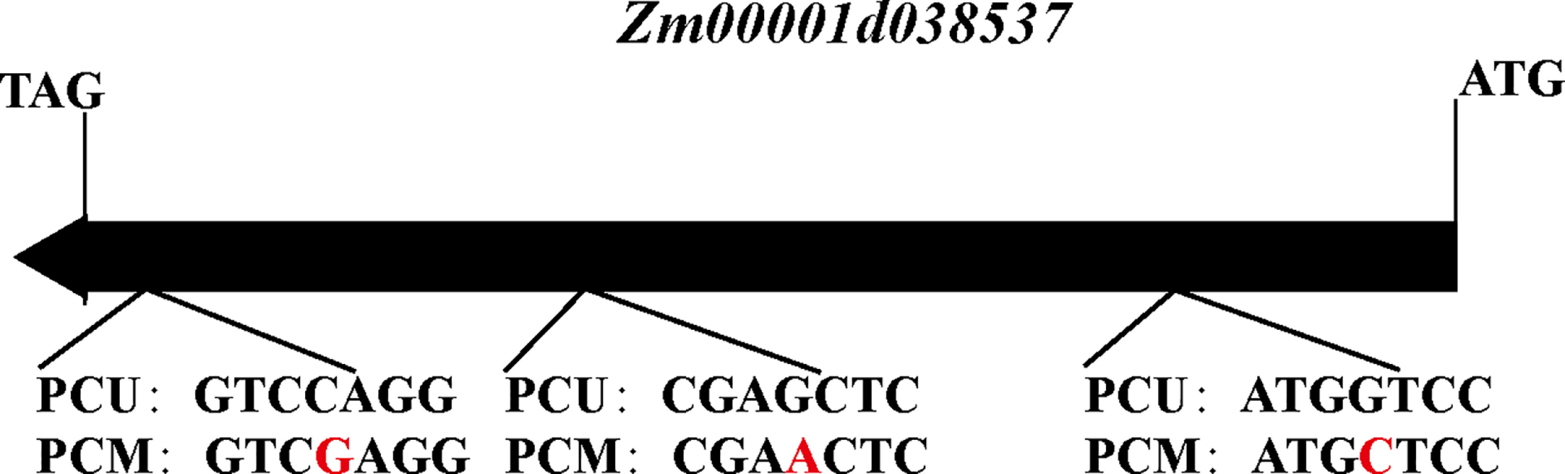
The structure of the *Zm00001d038537* between PCU and PCM. Red letters indicate SNP. The direction of the arrow represents the direction of transcription.

In addition, our annotation of genes in the *qTBN6.06-1* interval revealed a gene encoding the F-box structural domain *Zm00001d038537*. Members of the F-box family of proteins can play roles in forming Skp1-Cullin-F-Box (SCF) structural complexes that ubiquitinate specific proteins and thereby promote their degradation, which is similar to processes that can also occur in the IAA metabolic pathway. The KEGG enrichment results provided evidence to indicate that phytohormone signaling, particularly IAA signaling, might contribute to the observed differences in TBN, as well as differences in the IAA content of parent tassels. Although *Zm00001d038537* was not differentially expressed in the parents, we inferred that *Zm00001d038537* might be a candidate gene responsible for TBN differences. Cloning and sequencing of this gene in both parents revealed three SNPs, the first and third of which encoded different amino acids (**Figure 7**), resulting in different encoded proteins. These differences were found to influence IAA signaling and led to differences in the number of male spike branches. Consequently, *Zm00001d038537* was included as a candidate gene.

## 4 Discussion

In this study, in which we sought to gain insights into the genetic regulation of tassel development in maize, we used the unbranched parent PCU and multi-branched parent PCM, two high-generation sister lines with high background similarity, to construct a genetic linkage map with a small distribution of markers on a single chromosome. PCU was characterized by an absence of tassel branching under different environmental conditions, thereby indicating that the branching trait in this line is not subjected to environmental control. However, on the basis of our field observations and analysis of natural seed setting rates, we identified no significant differences between the PCU and PCM lines.

Thirteen QTLs were identified on chromosomes 3, 6, and 7. Maize chromosomes 3 and 7 are known hotspots for QTL localization, containing genes associated with tassel development, including *Lg2*, nana plant 1 *(Na1)*, *Ba1*, *Sid1*, *Tsh4*, and *Ra3* (Walsh and Freeling, 1999; Satoh-Nagasawa et al., 2006; Chuck et al., 2007; Chuck et al., 2008; Gallavotti et al., 2008; Hartwig et al., 2011; Phillips et al., 2011). Previously, Chen et al. (2014) constructed an F_2_ population comprising 708 individual strains and detected seven TBN-related QTLs, among which the location results obtained for chromosome 3 coincided with *qTBN-3-3*. Moreover, Wang performed similar analyses on the progeny of natural and doubled-haploid populations, and accordingly identified 12 loci (distributed on chromosomes 1, 2, 3, 4, 6, and 7) consistent with multiple environments. Among these, the QTLs located on chromosome 3 overlap with the those observed in the current study. Moreover, our transcription data also revealed notable differences in the predicted candidate gene *Zm00001d042794* (Wang et al., 2019). Therefore, we identified a new QTL (*qTBN-3-1*) on chromosome 6, which coincides with *Lg2*, a gene that has been established to control leaf angle and TBN in maize. In addition, *qTBN-7-3* was found to harbor *Tsh4* (which is associated with tassel development) and *Ra3* (which is known to regulate the number of tassel branches), which coincide respectively with the *qBTBN7-1* and *qXTBN7-1* loci mapped by Wang et al., 2018.

In this study, the QTL identified on chromosome 6 accounted for 9.39% to 40.77% of the phenotypic variation and was detected in different environments. Similarly, previous studies have identified 14 TBN-related loci on chromosome 6, classified into seven groups on the basis of their physical locations (Li et al., 2019). However, these loci contributed to less than 10% of the observed phenotypic differences and did not coincide with the results of the present study. Furthermore, although the QTLs localized in the present study overlap with those reported by Yi et al. (2018), the distribution range detected by Yi et al. was relatively large, making it difficult to directly compare the respective QTLs.

**Figure 8:**
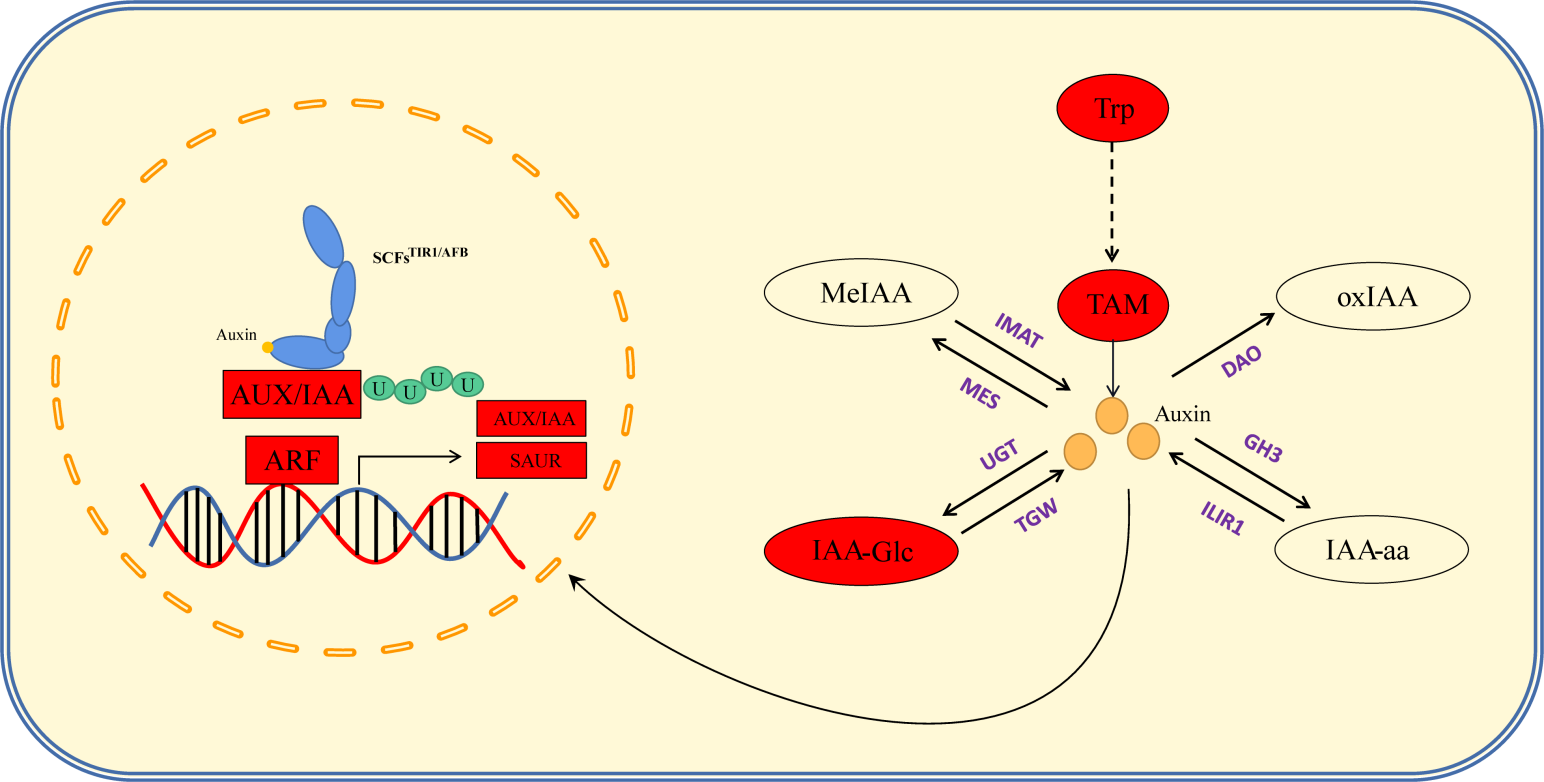
Auxin anabolism and signal regulation pathway. The red box indicates that the gene expression level of PCU is higher than that of PCM, and the red oval indicates that the hormone content of PCU is higher than that of PCM. Purple stands for key enzymes in anabolism.

Auxin is an important hormone involved in plant growth and development and is one of several hormones known to influence tassel branching in plants. *Vt2* (vanishing tassel 2) (Phillips et al., 2011) and *Spi1* (sparse inflorescence 1) (Gallavotti et al., 2008) have been identified as genes involved in auxin synthesis, the mutation of which has been found to coincide with a reduction in maize TBN, thereby providing evidence to indicate that these genes are involved in the initiation and growth of the axillary meristem during maize tassel development. In the present study, we combined our hormone determination results with the findings of KEGG pathway enrichment analysis to elucidate the regulatory pathways from hormones to response genes (**Figure 5**; **Table S3**). Auxin synthesis pathways can be divided into two main categories, namely, tryptophan (TRP)-dependent and TRP-independent (Mano and Nemoto, 2012), and the pathways involved in IAA metabolism primarily include IAA oxidation and methylation, resulting in the formation of conjugates with polysaccharides and amino acids (Zhao, 2012). In this study, we assessed the auxin synthesis pathway by synthesizing IAA *via* TAM, which entailed analyses of the contents of TRP, TAM, IAA, IAA-Glu, IAA-glc, IAA-ASP, MeIAA, and oxIAA. By mapping the auxin anabolic and gene response pathways based on KEGG results, we found that the contents of TRP and TAM in the PCU line were significantly higher than those in the PCM line, whereas IAA contents in the two lines was relatively similar, with only slightly higher levels being detected in PCU. Among the assessed IAA metabolites, only the content of IAA-glc was markedly higher in PCU than in PCM. On the basis of these observations, we thus infer that whereas larger amounts of IAA are synthesized in PCU, a large proportion is stored in the form of IAA-glc, and thus the levels of IAA detected in the two the parental lines tend to be similar (**Figure 8**).

Auxin signal transduction is regulated by multiple genes, and IAA enters the cell nucleus through the amino acid permease input carrier protein (auxin resistant-like aux1, *AUX/LAX*) (Swarup and Péret, 2012). In response to low IAA concentrations, auxin/indole-acetic acid genes (*AUX/IAA*) form a heterodimer with *ARFs* (Enders and Strader, 2015), thereby inhibiting the expression of downstream genes. Conversely, when present at high concentrations, IAA combines with transport inhibitor resistant 1/auxin signaling F-box (TIR1/AFB) and AUX/IAA. TIR1/AFB participates in the formation of SCF E3 ubiquitin ligase (Fendrych et al., 2018), resulting in the polyubiquitination of AUX/IAA, subsequent degradation *via* 26S proteasome, and the release of *ARF* inhibition. This also promotes or inhibits the expression of downstream IAA response genes [*AUX/IAA*, *Gh3*, and *SAUR* (small auxin upregulated RNA)]. We speculate that the slightly higher levels of IAA detected in PCU may have resulted in the degradation of AUX/IAA, and a correspondingly enhanced expression of *ARFs*, *AUX/IAA*, and *SAUR*, thus regulating tassel development and branching. Furthermore, given that we detect no significant difference in the expression of the IAA polar transport gene peptidylprolyl *cis*/*trans* isomerase, NIMA-interacting 1 (*PIN1*) between the two parental lines, it is reasonable to assume that the regulation of tassel branching is unrelated to the polar transport of auxin (**Figure 8**).

Tassel development and branching are assumed to be regulated by multiple hormones. In this regard, CK can alleviate apical dominance and promote lateral branch growth (Bangerth, 1994; Turnbull et al., 1997; Tanaka et al., 2006; Hoyerova and Hosek, 2020). However, CK activity is often regulated by auxin, which in turn promotes the growth of lateral buds by promoting the polar transport of IAA in stems and upregulating IAA synthesis in buds. Furthermore. it has been demonstrated that *ARF19* can inhibit the expression of isopentenyl transferases (IPTs) and control the synthesis of CTK (Li et al., 2006). Although in the present study, we detected the upregulated expression of certain ARFs, we observed no significant differences in IPT gene expression or tZ content in the sister lines studied. Moreover, whereas we recorded high levels of N6-isopentenyl-adenine-9-glucoside (iP9G) content in PCM, this compound was not detected in PCU, and we accordingly speculate that iP9G could be involved in the regulation of TBN (**Figure S3**).

## 5 Conclusion

In this study, we used the sister maize lines PCU and PCM, characterized by significant differences in tassel branch number, as parents to produce an F_2_ population, and applied a genetic microarray to genotype the parents and F_2_ population, and to construct an associated genetic linkage map. On the basis of phenotypic and genotypic data, we identified two major QTLs, *qTBN6.06-1* and *qTBN6.06-2*, on chromosome 6. RNA-seq analysis of material collected at three stages of tassel development revealed that DEGs were enriched in amino acid metabolism and phytohormone signaling. Additionally, we established that levels of IAA, IAA-glc, TRP, and TAM were higher in PCU than in PCM, whereas in contrast, PCM was characterized by higher levels of 5DS. By combining our localization results and transcriptome data, we able to identify five candidate genes that putatively contribute to the regulation of tassel branching. Our findings in this study provide a theoretical basis that will potentially contribute to improving tassel traits in maize breeding.

## Data availability statement

The data presented in the study are deposited in the SRA repository: https://www.ncbi.nlm.nih.gov/sra/PRJNA998913.

## Author contributions

SR: Investigation, data curation, validation, and writing—original draft. HS: Review and editing. QY: Data curation, methodology, formal analysis, software. LYim: Formal analysis and editing. ZX: Investigation and formal analysis. LYin: Investigation. LXih: Formal analysis. LF: Writing—review and editing. DM: Investigation. LXia: Conceptualization, writing—review, editing and funding acquisition. All authors contributed to the article and approved the submitted version.
