# Supplementary material for: Identification of QTLs and their candidate genes for the number of maize tassel branches in F2 from two higher generation sister lines using QTL mapping and RNA-seq analysis
Source: Front Plant Sci. 2023 Aug 13;14:1202755. doi: 10.3389/fpls.2023.1202755 (PMC10460468; doi:10.3389/fpls.2023.1202755)
Supplement: Supplementary file 1 [file DataSheet_1.docx]

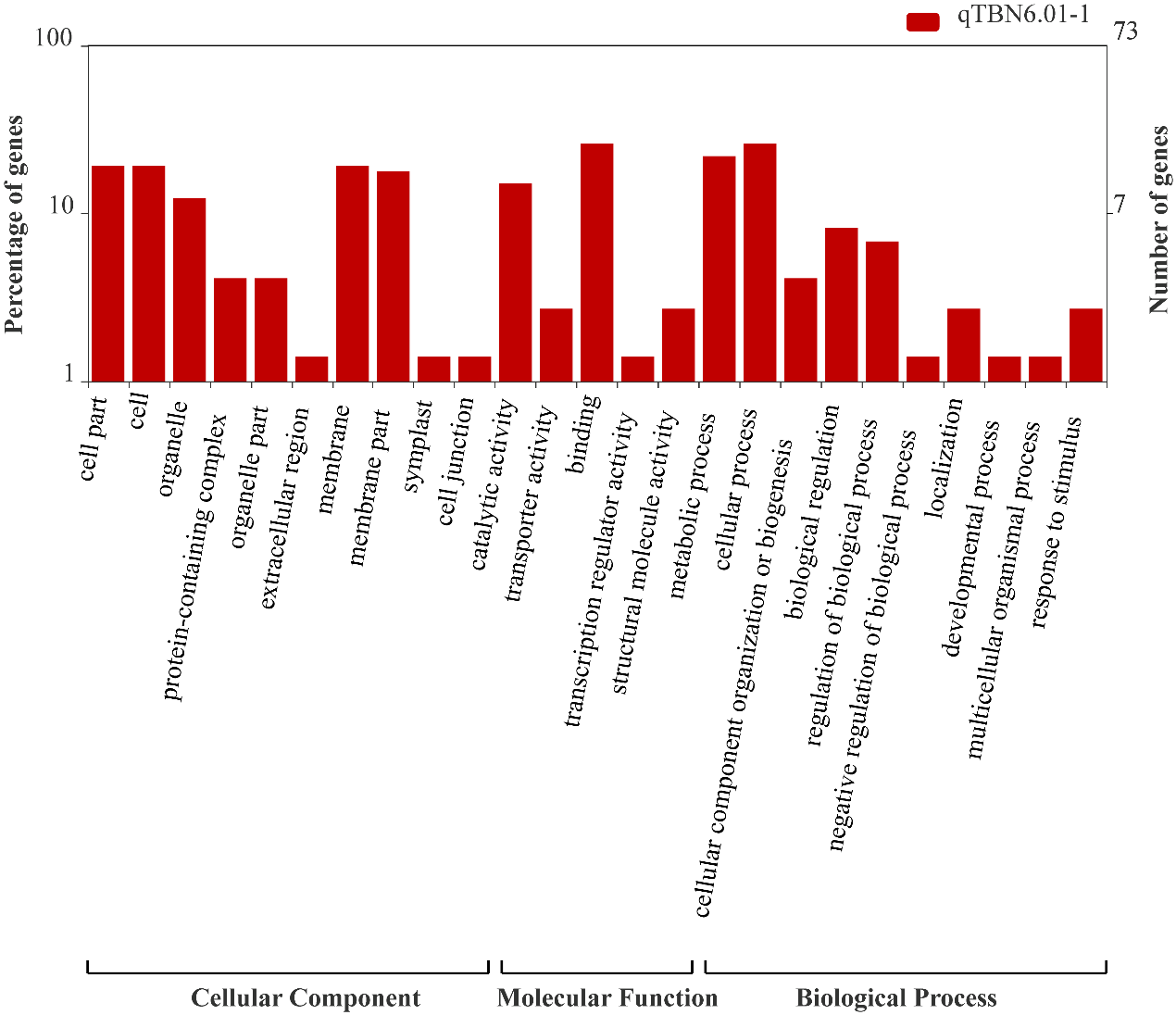


**Figure S1**. GO term analysis of interval genes in qTBN6.01-1 using WEGO 2.0. The ruler on the left side indicates the number of genes contained in the entry as a percentage of the total number of genes, and the ruler on the right indicates the number of genes contained in the term.

F
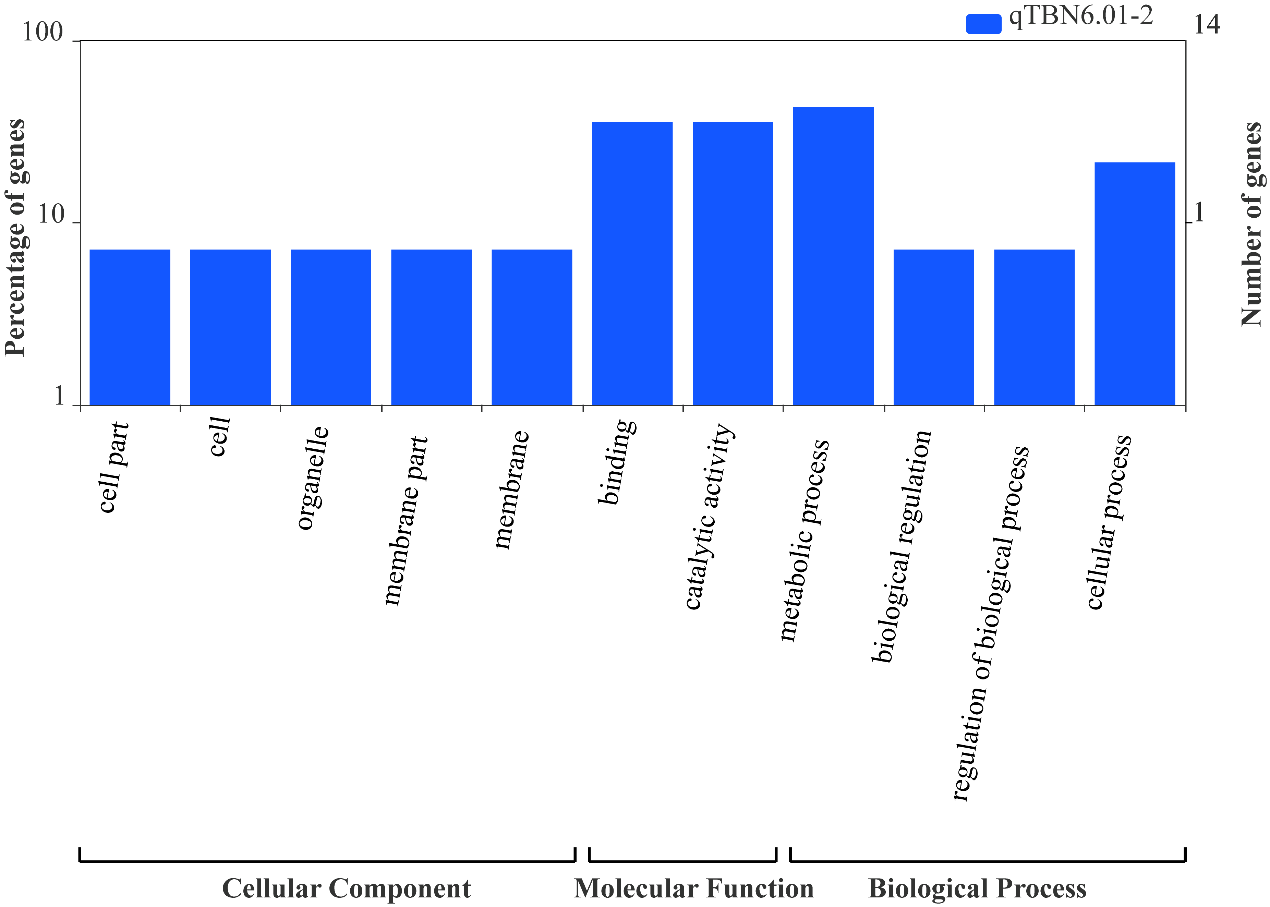
igure S2. GO term analysis of interval genes in qTBN6.01-2 using WEGO 2.0. The ruler on the left side indicates the number of genes contained in the entry as a percentage of the total number of genes, and the ruler on the right indicates the number of genes contained in the term.


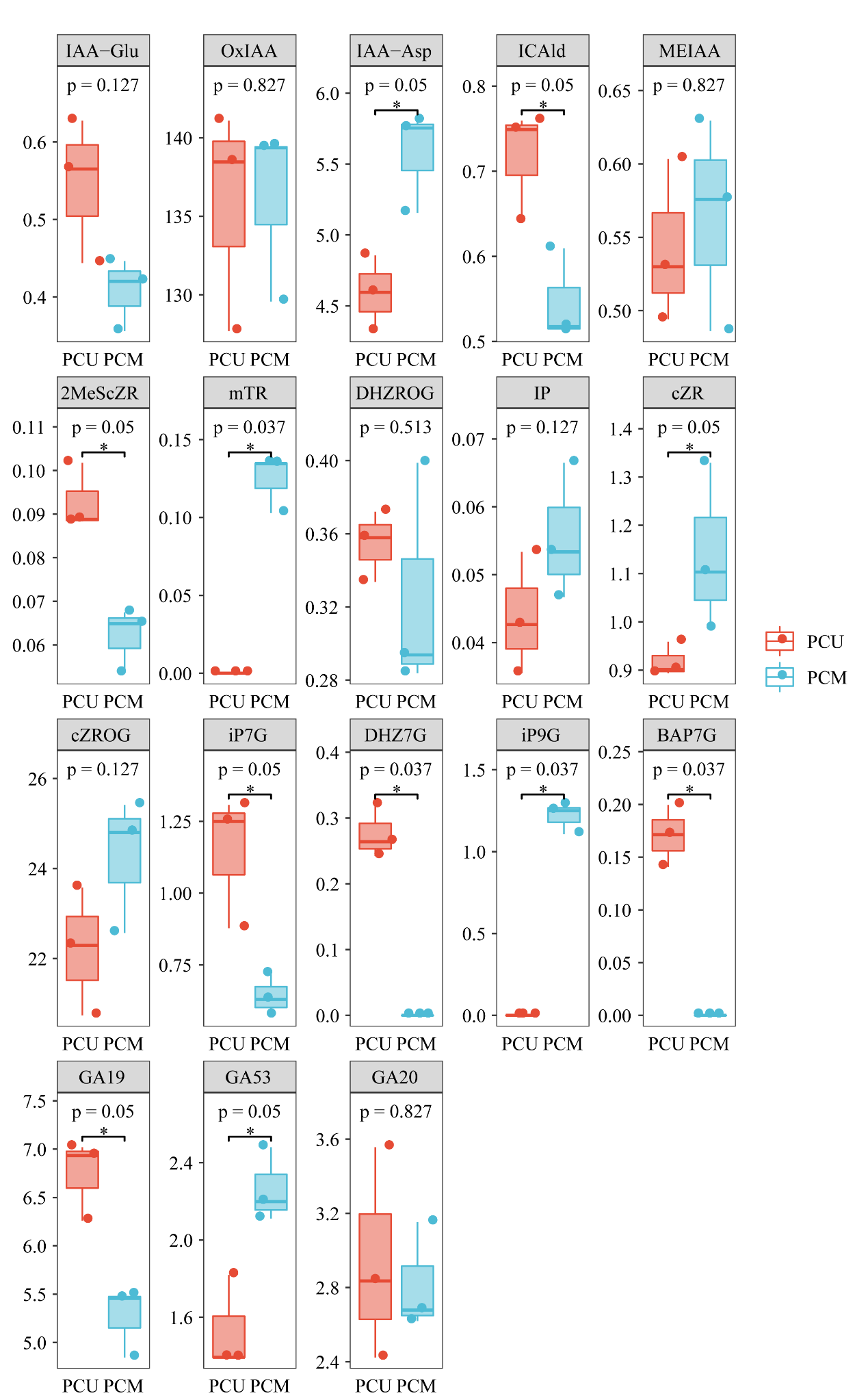
Figure S3. Different phytohormone contents of PCU and PCM in stage I. *P < 0.05


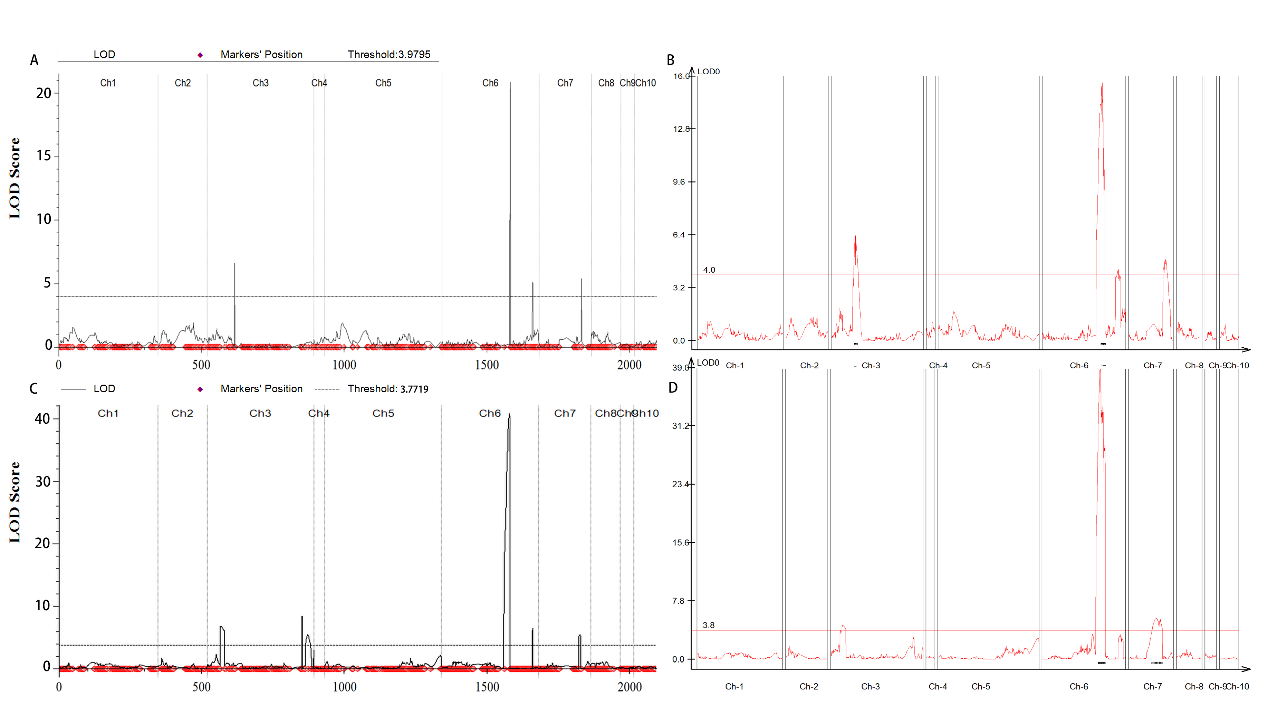


FigureS4: QTL localization of F_2_ and F_2:3_ by both ICIM and CIM methods. A: QTL localization of TBN in F_2_ by ICIM method. B: QTL localization of TBN in F_2_ by CIM method. C: QTL localization of TBN in F_2:3_ by ICIM method. D: QTL localization of TBN in F_2:3_ by CIM method.


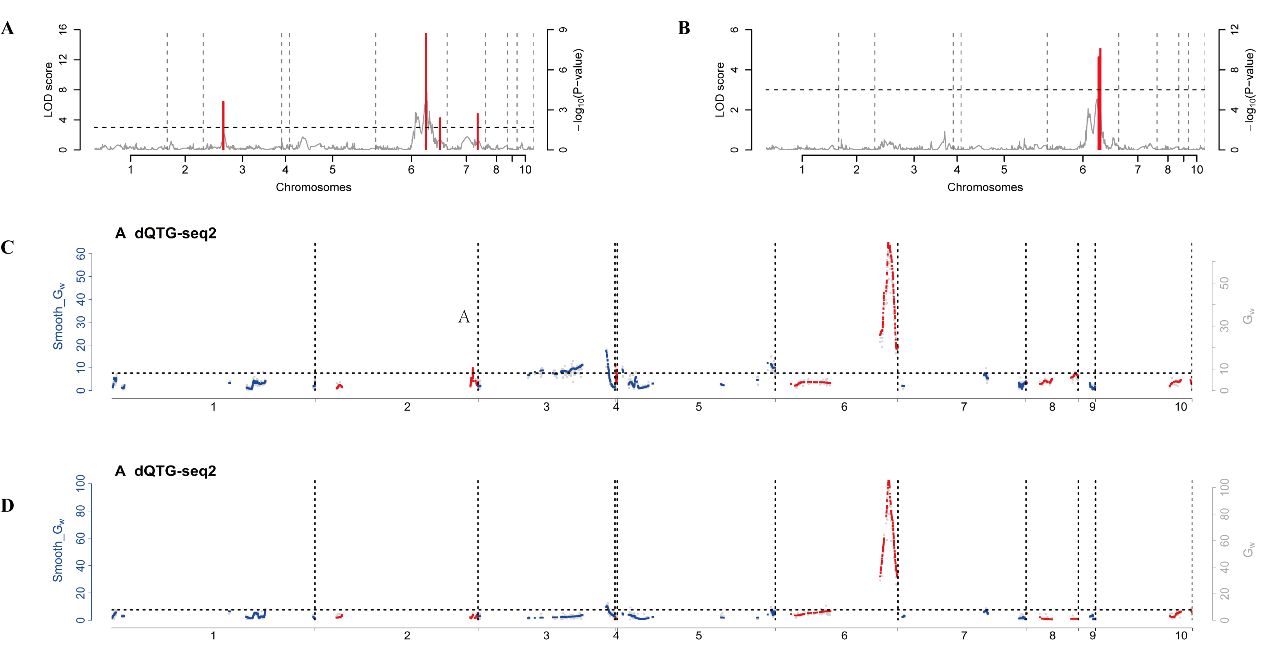


FigureS5: QTL localization and QTNs of F_2_ and F_2:3_ by both GCIM and dQTG-seq methods. A: QTL localization of TBN in F_2_ by GCIM method. B: QTL localization of TBN in F_2:3_ by GCIM method. C: QTNs of TBN in F_2_ by dQTG-seq method. D: QTNs of TBN in F_2:3_ by dQTG-seq method.
